# Perspectives of Rehabilitation Specialists on Telerehabilitation in Jordan: Knowledge, Attitudes, and Implementation Barriers

**DOI:** 10.1155/ijta/5394554

**Published:** 2026-05-24

**Authors:** Mohammad Al-Wardat, Abidal Aziz Almomani, Mohammad Yabroudi, Khader Almhdawi

**Affiliations:** ^1^ Department of Rehabilitation Sciences, Faculty of Applied Medical Sciences, Jordan University of Science and Technology, Irbid, Jordan, just.edu.jo

**Keywords:** implementation barriers, Jordan, knowledge and attitudes, rehabilitation specialists, telerehabilitation

## Abstract

**Background:**

Telerehabilitation (TR) has emerged as a promising approach to improving access to rehabilitation services, particularly in low‐ and middle‐income countries such as Jordan. However, successful implementation largely depends on rehabilitation specialists’ knowledge, attitudes, and perceived barriers.

**Aims:**

This study is aimed at exploring rehabilitation specialists’ perspectives on TR in Jordan, focusing on their knowledge, attitudes, current use, and perceived implementation barriers.

**Methods:**

A cross‐sectional study was conducted among rehabilitation specialists including physical therapist, occupational therapist, and speech and language therapist practicing in Jordan. Participants completed a validated 27‐item Rehabilitation Specialists’ Knowledge, Attitudes, and Barriers to TR Questionnaire. Descriptive statistics were used to summarize participants’ demographic characteristics and their responses related to TR knowledge, attitudes, and perceived barriers.

**Results:**

A total of 300 rehabilitation specialists (mean age = 28.6 ± 6.1 years; 51% female) were included in the analysis, comprising equal numbers of physiotherapists, occupational therapists, and speech and language therapists. Overall, 81% of participants reported awareness of TR, and 57% indicated that TR was currently used in their workplace. Despite the generally favorable perceptions—59% considered TR socially somewhat acceptable and 75% believed it could save time, effort, or costs—actual engagement remained limited, with 35% reporting awareness without prior use and only 14% reporting regular use. Technological reliability and validity were endorsed by 78% and 72% of respondents, respectively. However, only 36% agreed that TR was comparable to face‐to‐face care. The most frequently reported barriers included limited public awareness (84%), technical issues such as poor internet connection (80%), provider willingness (74%), staff skill limitations including lack of training (71%), data privacy and security concerns (71%), location of healthcare institutions (69%), increased workload (69%), and lack of user‐friendly software (61%). Early‐career clinicians (< 5 years of experience), who constituted 67% of the sample, demonstrated greater openness toward TR compared with more experienced practitioners.

**Conclusion:**

Rehabilitation specialists in Jordan showed readiness to adopt TR. However, significant structural, educational, and cultural barriers appeared to hinder its widespread implementation.

## 1. Introduction

Telerehabilitation (TR), a specialized branch of telemedicine, refers to the delivery of rehabilitation services at a distance through information and communication technology (ICT) [1]. It has gained increasing global attention to improve access to rehabilitation care, enhance patients’ outcomes, and reduce healthcare costs while maintaining quality comparable to in‐person services. Previous studies suggested that TR can be effective for a range of clinical populations, including neurological, musculoskeletal, and cardiorespiratory conditions [2–5]. Specifically, TR has shown efficacy in improving physical function, quality of life, and patients’ satisfaction. In addition, TR offers benefits in terms of cost and accessibility [6]. Despite its potential, the implementation of TR in many developing regions worldwide remains difficult. Research conducted across various countries revealed that TR adoption is strongly influenced by healthcare providers’ levels of knowledge and awareness, as well as their attitudes toward remote service delivery [7–9]. For example, a recent study conducted among Tunisian rehabilitation professionals has shown that while most participants were possibly aware of TR concepts, actual use in clinical practice was relatively low [10]. The study identified several TR application obstacles, including limited training, technical challenges, and infrastructural constraints.

Healthcare providers’ attitudes toward technology and willingness to adopt it are commonly shaped by their perceptions of its usefulness, compatibility with professional roles, and their confidence in using it [11]. Evidence from broader telemedicine research highlighted that even when attitudes toward technology were positive, limited knowledge and lack of targeted education correlated with suboptimal uptake of telemedicine services [12]. In Jordan, telemedicine has been explored in general applications such as chronic disease management [8, 13]. These studies identified different major challenges, including healthcare professionals’ limited awareness, technological barriers, and resistance to change [8, 13]. Qualitative research focused on telemedicine for cardiovascular care, for example, noted several barriers experienced by healthcare professionals, including concerns about workflow integration and practical implementation issues [14].

However, specific evidence on knowledge, attitudes, and perceived barriers related explicitly to TR among Jordanian rehabilitation care providers is still unclear. To the best of our knowledge, the existing literature lacks a detailed investigation of the knowledge, attitudes, and perceived barriers to TR among healthcare providers in Jordan. In addition, the rehabilitation community in Jordan lacks specific guidelines related to the implementation and practice of TR. Previous studies conducted in the Middle East and North Africa and other low‐resource settings have highlighted several challenges [15–18]. Examples of these challenges include inadequate ICT infrastructure, a lack of local guidelines, and workforce training needs [15, 19].

Overall, the burden of disability is gradually increasing nationally and internationally [20]. In addition, the transition toward telemedicine is a demand that has been accelerated by the COVID‐19 pandemic, highlighting the need for alternative service delivery models [21]. Given the strategic importance of sustainable access to rehabilitation services, it is critical to understand how healthcare providers in Jordan perceive and engage with TR. Therefore, the aim of this study was to assess the knowledge, attitudes, and perceived barriers toward TR among rehabilitation care providers in Jordan. We hypothesized that rehabilitation professionals will demonstrate an overall positive knowledge and attitudes toward TR. Secondarily, we hypothesized that significant technical, organizational, and training‐related barriers may limit TR implementation. The results of this study may help reshape health policy, education, and implementation strategies that will possibly support effective TR services in Jordan.

## 2. Materials and Methods

### 2.1. Study Design, Settings, and Participants

This cross‐sectional study targeted rehabilitation professionals in Jordan, including physiotherapists (PTs), occupational therapists (OTs), and speech and language therapists (SLTs). The inclusion criteria were (1) therapists actively engaged in rehabilitation practice in Jordan, (2) employment in public or private hospitals or clinics and at least 1 year of experience, and (3) proficiency in both Arabic and English. Exclusion criteria included retirement, practicing outside Jordan, and being suspended or deemed unfit to practice. The target sample size was 300 participants, based on MacCallum’s guidelines for cross‐sectional studies, which suggest that a sample size of 300 or more is considered excellent [22].

### 2.2. Study Instrument

A structured, self‐administered questionnaire was used to collect data. The instrument was adapted from a previously validated English questionnaire assessing rehabilitation professionals’ knowledge, attitudes, and barriers related to TR, originally developed by rehabilitation researchers in Saudi Arabia [23]. For the current study, the questionnaire was culturally adapted for use in Jordan and refined through expert review and pilot testing. The final instrument consisted of 27 items, organized into four domains, consistent with the analytical structure used in this study.

#### 2.2.1. Domain 1: Sociodemographic and Professional Characteristics (Nine Items)

This domain captured essential participant background information, including gender, age, region of residence, profession (PT, OT, and PLS), workplace setting, patient population served, educational level, monthly income, and years of professional experience. These items were descriptive and were reported in means/standard deviations or counts/percentages.

#### 2.2.2. Domain 2: Knowledge of TR

This domain assessed participants’ knowledge and perceptions of TR, including 11 questions that assessed awareness, workplace availability, self‐reported level of knowledge and use, scope of services, social acceptability, current use in practice, tools used for delivery, perceived benefits (time, effort, and cost efficiency), patient acceptance, need for training, and perceived cost of required equipment. Items were measured using dichotomous (yes/no), multiple‐choice, and Likert‐type responses. For scoring, each item was dichotomized and assigned 1 point for responses reflecting adequate knowledge (e.g., awareness of TR, workplace availability, reporting use or good knowledge, recognizing broader clinical applications, perceiving it as acceptable, acknowledging its benefits, and identifying patient acceptance) and 0 for negative or uncertain responses. The total composite score ranged from 0 to 11, with higher scores indicating greater knowledge of TR.

#### 2.2.3. Domain 3: Attitudes Toward TR (Six Items)

This domain evaluated participants’ attitudes toward TR, including perceptions of its reliability and clinical utility, perceived benefits for patients and healthcare providers, quality comparability to face‐to‐face sessions, and expectations regarding cost coverage by government or insurance. Items were assessed using statements scored by a Likert scale (ranging from strongly agree/significant to strongly disagree/not significant) and dichotomous responses (yes/no). For scoring, responses were dichotomized, with 1 point assigned to positive attitudes (e.g., “strongly agree/agree,” “strongly significant/significant,” or “yes” for cost coverage) and 0 assigned to neutral or negative responses. The total score ranged from 0 to 6, with higher scores indicating more positive attitudes toward TR.

#### 2.2.4. Domain 4: Perceived Barriers Facing TR (One Item)

This domain assessed perceived barriers to the implementation of TR in Jordan through a single multiresponse question asking participants to identify potential challenges. Subitems included provider willingness, technical issues (e.g., poor internet connectivity), staff skill limitations (e.g., lack of training and limited clinical utility), high cost, location of the healthcare institution, data privacy and security concerns, lack of user‐friendly software, increased workload, and limited public awareness. Each subitem was measured using a dichotomous (yes/no) response format. For scoring, 1 point was assigned for each barrier endorsed (“yes”) and 0 for “no,” with total scores ranging from 0 to 9. Higher scores indicated greater perceived barriers to the use of TR.

The adapted questionnaire underwent pilot testing, which recruited 15 rehabilitation professionals to evaluate the survey clarity, cultural relevance, and feasibility. Minor refinements were made based on participants’ feedback. Reliability testing using Cronbach’s alpha demonstrated acceptable internal consistency across all domains, with alpha values exceeding 0.71. Content validity was established through review by three experts in rehabilitation and telehealth, who evaluated item relevance and contextual appropriateness prior to final deployment.

### 2.3. Procedure for Data Collection and Analysis

A convenience sampling strategy was employed to recruit participants through professional and workplace social media groups (e.g., WhatsApp, Facebook, and LinkedIn). This approach was selected due to the absence of a comprehensive national registry of rehabilitation professionals in Jordan, which limited the feasibility of probability‐based sampling methods. Social media platforms are widely utilized by healthcare professionals in Jordan and provide an efficient means to reach a geographically diverse sample across rehabilitation disciplines and practice settings. To facilitate this, the survey invitation was distributed across multiple professional groups to enhance diversity in the sample.

Data were entered and cleaned using Microsoft Excel. Statistical analyses were conducted using SPSS Version 26, with descriptive statistics used to summarize demographic characteristics and knowledge and attitude scores. The survey was administered in English. This decision reflects the educational background of healthcare providers in Jordan, as university‐level health science education is primarily delivered in English.

### 2.4. Ethics Statement

The study protocol was reviewed and approved by the Institutional Review Board of Jordan University of Science and Technology, Jordan (Approval No. 2023/160/31). All procedures were conducted in accordance with the principles of the Declaration of Helsinki. Electronic informed consent was obtained from all participants prior to survey completion. All respondents were provided with detailed study information prior to providing informed consent. Participation was voluntary, and no personal identifying information was collected.

## 3. Results

### 3.1. Participants’ Characteristics

A total of 379 rehabilitation specialists responded to the survey. After eligibility assessment and signing the informed consent, 79 responses were excluded due to incomplete questionnaires (*n* = 45), failure to meet the inclusion criteria (*n* = 22), or not currently practicing (*n* = 12). The final analysis included 300 participants who met all study eligibility criteria. The final sample comprised an equal number of PTs, OTs, and SLTs (*n* = 100 each), with a mean age of 28.6 ± 6.11 years. The majority of participants were early‐career professionals under 30 years of age (74%) and females (51%). Most resided in urban areas (61%) and held a bachelor’s degree (71%).

Participants worked across diverse settings, including public hospitals, private clinics, private and military hospitals, academic institutions, and freelance practice, reflecting a broad representation of the rehabilitation workforce in Jordan. Monthly income varied widely, with most earning between 300 and 500 JOD. The sample was predominantly composed of clinicians with less than 5 years of professional experience (67%), highlighting the relatively young and early‐career nature of the workforce included in this study. These demographic characteristics suggest that the findings largely reflect the perspectives of early‐career, urban‐based rehabilitation specialists, which may have implications for their familiarity with, and openness to, emerging technologies such as TR (Table 1).

**Table 1:** Sociodemographic and professional characteristics of the participants (*N* = 300).

Characteristic	Category	*N*(%)
Profession	Physiotherapists	100 (33.3)
	Occupational therapists	100 (33.3)
	Speech and language therapists	100 (33.3)

Age (years)	Mean ± SD	28.6 ± 6.11
	23–30	222 (74.0)
	31–35	30 (10.0)
	36–40	33 (11.0)
	> 40	15 (5.0)

Gender	Female	153 (51.0)
	Male	147 (49.0)

Area of residence	Urban	183 (61.0)
	Rural	117 (39.0)

Highest educational qualification	Diploma (2 years)	33 (11.0)
	Bachelor’s degree	213 (71.0)
	Master’s degree	54 (18.0)

Workplace setting	Public hospitals	99 (33.0)
	Private hospitals	33 (11.0)
	Military hospitals	27 (9.0)
	Private clinics	78 (26.0)
	Freelance/private sessions	36 (12.0)
	Academic staff	27 (9.0)

Monthly income (Jordanian Dinar, JOD)	< 300	81 (27.0)
	300–400	99 (33.0)
	401–500	69 (23.0)
	501–600	12 (4.0)
	> 600	39 (13.0)

Primary patient population	Adults	84 (28.0)
	Pediatrics	30 (10.0)
	Geriatrics	9 (3.0)
	Psychiatric	9 (3.0)
	Mixed populations	168 (56.0)

Years of professional experience	< 5 years	201 (67.0)
	5–10 years	66 (22.0)
	11–15 years	18 (6.0)
	> 15 years	15 (5.0)

### 3.2. Knowledge of TR

Most participants (81%) reported good awareness of TR, though only 43% indicated that it was currently available in their workplace. Actual engagement in TR varied: While 33% used it intermittently and 14% reported good knowledge with regular use, a substantial proportion (35%) were aware but had never applied it in practice, and 18% were unaware of the technology. In terms of services offered, just over half of participants (57%) reported access to a comprehensive range of TR services—including consultation, prescription, therapy delivery, monitoring, evaluation, and follow‐up—while the remainder reported more limited offerings. Social acceptability of TR among rehabilitation specialists was generally high, with 93% rating it at least somewhat acceptable. Social media platforms were the most used tools (60%), followed by phone or SMS (29%) and email (11%). Additionally, three‐quarters of respondents (75%) believed that TR could save time, effort, or costs, highlighting its perceived efficiency benefits. Knowledge of TR results is demonstrated in Table 2.

**Table 2:** Participants’ responses on knowledge and perceptions of telerehabilitation (*N* = 300).

Item	Response	*N*(%)
Awareness of telerehabilitation	Yes	242 (81)
	No	58 (19)

Availability of telerehabilitation at workplace	Yes	129 (43)
	No	171 (57)

Self‐reported knowledge of telerehabilitation	Aware but not used	107 (35)
	Aware and used intermittently	98 (33)
	Good knowledge and used regularly	42 (14)
	Not aware of the technology	53 (18)

Services that can be delivered via telerehabilitation	Consultation, prescription, therapy delivery, and monitoring	170 (57)
	Comprehensive services (consultation, prescription, complex therapy delivery, monitoring, evaluation, follow‐up, rehabilitation care)	130 (43)

Social acceptability of telerehabilitation in Jordan	Extremely acceptable	40 (13)
	Very acceptable	64 (21)
	Somewhat acceptable	175 (59)
	Not acceptable	21 (7)

Use of telerehabilitation in current practice	Yes	171 (57)
	No	129 (43)

Tools used for telerehabilitation delivery	Social media platforms	180 (60)
	Phone calls/SMS	88 (29)
	Email	32 (11)

Telerehabilitation can save time, effort, and/or cost	Yes	225 (75)
	No	21 (7)
	Do not know	54 (18)

Perceived patient acceptance of telerehabilitation in Jordan	Yes	167 (56)
	No	52 (27)
	Do not know	81 (17)

Need for training in telerehabilitation	Yes	205 (68)
	No	60 (20)
	Do not know	35 (12)

Telerehabilitation requires expensive equipment	Yes	112 (38)
	No	130 (43)
	Do not know	58 (19)

### 3.3. Attitudes Toward TR

The majority of participants considered TR technology to be reliable (78%) and valid (72%), reflecting general confidence in its technical feasibility. Participants also recognized its potential benefits: Two‐thirds (66%) believed TR could effectively support diverse patient populations, while slightly more (71%) saw advantages for rehabilitation professionals in their clinical practice. When comparing TR to traditional in‐person sessions, only about one‐third of respondents (36%) considered the two approaches equivalent, with a similar proportion expressing neutrality and disagreement. Regarding financial feasibility, just over half of participants (55%) anticipated that TR services would be financially supported by the government and/or insurance programs. Attitudes toward TR results are demonstrated in Table 3.

**Table 3:** Participants’ understanding and attitudes toward telerehabilitation (*N* = 300).

Item	Response	*N*(%)
Reliability of telerehabilitation technology	Strongly significant	59 (20)
	Significant	175 (58)
	Not significant	54 (18)
	Strongly not significant	12 (4)

Validity of telerehabilitation technology	Strongly significant	45 (15)
	Significant	172 (57)
	Not significant	66 (22)
	Strongly not significant	17 (6)

Patients can benefit from telerehabilitation	Strongly agree	54 (17)
	Agree	156 (50)
	Neutral	87 (28)
	Disagree	10 (3)
	Strongly disagree	5 (2)

Healthcare providers can benefit from telerehabilitation	Strongly agree	55 (18)
	Agree	158 (53)
	Neutral	64 (21)
	Disagree	18 (6)
	Strongly disagree	5 (2)

Telerehabilitation offers similar benefits to face‐to‐face sessions	Strongly agree	30 (10)
	Agree	79 (26)
	Neutral	93 (31)
	Disagree	75 (25)
	Strongly disagree	22 (8)

Likelihood of cost coverage by government or insurance	Yes	165 (55)
	No	135 (45)

### 3.4. Perceived Barriers Facing TR

Participants identified multiple barriers to the implementation of TR in Jordan, with limited public awareness emerging as the most frequently reported challenge (84%). Technical issues, including poor internet connectivity, were highlighted by 80% of respondents, while provider willingness and staff skill limitations—including insufficient training—were reported by 74% and 71%, respectively. Data privacy and security concerns were also noted by 71% of participants. Other commonly cited barriers included institutional location and increased workload (69%), as well as lack of user‐friendly software (61%) and high cost (52%). Perceived barriers facing TR are listed in Table 4.

**Table 4:** Reported barriers to the use of telerehabilitation in Jordan (*N* = 300).

Barrier	Yes *N*(%)	No *N*(%)
Provider willingness	221 (74)	79 (26)
Technical issues (e.g., poor internet connectivity)	240 (80)	60 (20)
Staff skill limitations (e.g., lack of training and limited clinical utility)	212 (71)	88 (29)
High cost	157 (52)	143 (48)
Location of healthcare institution	208 (69)	92 (31)
Data privacy and security concerns	213 (71)	87 (29)
Lack of user‐friendly software	183 (61)	117 (39)
Increased workload	207 (69)	93 (31)
Limited public awareness of telerehabilitation in Jordan	251 (84)	49 (16)

## 4. Discussion

This cross‐sectional descriptive study is aimed at assessing the knowledge, attitudes, and perceived barriers to TR among rehabilitation professionals in Jordan. To our knowledge, this is the first study conducted in Jordan providing comprehensive insights of TR as perceived by rehabilitation professionals. Overall, the findings demonstrated a relatively high level of awareness and positive perceptions of TR. However, practical implementation remains suboptimal, reflecting a set of structural, technical, and workforce‐related challenges. These study results provided foundational evidence to inform policy, education, and implementation strategies that support effective TR services in Jordan and similar developing countries.

A substantial proportion of participants (81%) reported being aware of TR, and more than half (57%) indicated that they use TR services in their practice. It is important to note that, in the Jordanian context, most therapists who reported using TR relied on basic and nonspecialized tools. These tools primarily include video calls and phone‐based communication, rather than structured or dedicated TR platforms. These findings suggest that TR has been gaining recognition within the Jordanian rehabilitation workforce, particularly among younger professionals as supported by this study’s mean age. Similar trends have been reported in other low‐ and middle‐income countries. Early‐career clinicians in these settings tend to show greater acceptance of telemedicine innovations due to their higher familiarity with technology and mobile platforms [24–26]. Despite this awareness, only 14% of participants reported good knowledge and regular use of TR. These results suggest a clear gap between conceptual knowledge and consistent clinical application.

In terms of perceived utility, participants generally expressed confidence in the potential of TR. These findings are in line with existing literature demonstrating the feasibility and effectiveness of TR across a range of rehabilitation conditions, including neurological, musculoskeletal, and chronic disease management [2–5]. Studies from high‐income settings have shown that TR can yield outcomes comparable to face‐to‐face interventions, particularly for follow‐up care, patient education, and home‐based exercise programs [6, 27]. The current findings suggest that Jordanian rehabilitation professionals share similar confidence in the technology’s potential, even though the local infrastructure and regulatory frameworks may not yet fully support its widespread adoption.

Participants of this study recognized multiple clinical applications of TR, with over half reporting that it could be used for a broad range of services, including consultation, therapy delivery, monitoring, evaluation, and follow‐up [1, 6]. Moreover, 75% of respondents believed that TR could save time, effort, and/or cost, reinforcing its perceived efficiency benefits for both providers and patients. Therapists perceived social acceptance of TR in Jordan was generally high, as more than 59% rated it as at least somewhat acceptable. However, perceptions of patient acceptance were slightly less assertive, as only 56% believed that patients would accept TR services. This discrepancy between provider acceptance and perceived patient readiness has also been reported in previous studies [28–30]. This finding may reflect concerns related to digital literacy, cultural preferences for in‐person care, and trust in remote healthcare services [29, 30]. Compared to developed western countries, these challenges are more prominent in Middle Eastern contexts, where face‐to‐face interaction is traditionally valued in healthcare delivery [31].

Despite the general positive attitudes observed in this study, several barriers to the implementation of TR in Jordan were identified. The most frequently reported barriers included limited public awareness (84%), technical issues such as poor internet connectivity (80%), provider willingness (74%), staff skill limitations and lack of training (71%), and concerns related to private data confidentiality (71%). These barriers are consistent with findings from regional and international studies, which highlighted infrastructure limitations, insufficient training, and unclear legal and ethical frameworks as major challenges to TR adoption [26, 32].

Training has also emerged as a critical need identified in this study. About 68% of participants reported that formal training is necessary for effective TR implementation. This finding highlights the importance of integrating TR competencies into undergraduate curricula, continuing professional development programs, and institutional training initiatives [33, 34]. Without adequate training, clinicians may lack confidence in clinical decision‐making, remote assessment, and the use of digital platforms.

Concerns regarding data privacy and security were also prominent, as reported by about 71% of this study participants. In addition, barriers such as increased workload (69%) and challenges related to the location of healthcare institutions (69%) further highlighted organizational TR constraints. These findings are well‐aligned with global discussions on telehealth governance and emphasize the need for clearer national guidelines, secure digital infrastructure, and appropriate regulatory frameworks to ensure safe and effective implementation of TR services [35–37].

### 4.1. Clinical Implications and Recommendations

The findings of this study suggest that despite high awareness and generally positive attitudes toward TR among rehabilitation professionals in Jordan, TR’s routine clinical use remains limited. Structured training programs that focus on practical TR competencies, including remote assessment, digital communication, and ethical and data security considerations, are highly needed. The predominant use of basic tools such as video and phone calls suggests that these modalities are currently the most feasible and accessible options within the local Jordanian context and similar developing countries. Therefore, efforts should prioritize optimizing the effective use of these widely available technologies to support TR clinical practices. While more advanced TR platforms may offer additional functionalities for standardized assessment and outcome monitoring, their adoption should be considered in terms of contextual feasibility, resource availability, and user readiness. Concerns regarding patient readiness and digital literacy suggest that TR should be implemented through carefully designed, patient‐centered approaches. Hybrid care models that combine remote and in‐person services may be more appropriate to start with in Jordan than rushing into TR as a definite replacement for face‐to‐face rehabilitation. The readiness of early‐career clinicians offers a valuable opportunity to advance TR through targeted training and clinical leadership. Overall, successful integration of TR into routine practice will require coordinated efforts to strengthen training, infrastructure, and institutional support.

### 4.2. Limitations

Several limitations should be acknowledged. The data were collected through a self‐reported questionnaire, which may be subjective in response to social and demographic bias. The survey was administered in English, which, although consistent with the educational background of Jordanian rehabilitation professionals, may still have influenced the interpretation of certain items. Moreover, the survey used in this study has only been previously applied in a single preliminary study, and its psychometric properties are not fully established, which may limit the reliability and generalizability of the findings. Additionally, although the sample size was robust and included equal representation from the three rehabilitation disciplines, most participants were under 30 years of age and had fewer than 5 years of clinical experience. Although this study’s online sampling technique facilitated broad dissemination of the survey, it may have associated with self‐selection bias, as individuals with greater interest or familiarity with TR may have been more inclined to participate. This study is also limited by its reliance on descriptive statistical analyses, which restricts the ability to draw inferential conclusions or determine statistically significant differences between subgroups (e.g., based on experience level or professional background). Although this approach is consistent with the exploratory nature of the study—given the limited existing evidence on TR in Jordan—it limits the strength of comparative interpretations. Future research employing inferential statistical methods (e.g., regression analyses or hypothesis testing) is warranted to better identify determinants and predictors of TR adoption and utilization. This study employed a convenience sampling approach using social media and professional networks, which may introduce selection bias and limit the representativeness of the sample. Participants who are more engaged with digital platforms or have a particular interest in TR may have been more likely to respond, potentially influencing the study findings. Additionally, the lack of a centralized sampling frame for rehabilitation professionals in Jordan constrained the ability to implement probability‐based sampling methods. Therefore, the generalizability of the results to the broader population of rehabilitation specialists should be interpreted with caution. Another important limitation of this study is the reliance on rehabilitation specialists’ perceptions regarding patients’ willingness and readiness to adopt TR. These views may not accurately represent the actual experiences, preferences, and barriers faced by patients or service users. Therefore, future research should directly engage patients to explore their perspectives, digital literacy, accessibility challenges, and acceptance of TR. Such patient‐centered evidence is essential for designing effective, inclusive, and contextually appropriate TR services and for informing successful implementation strategies.

## 5. Conclusions

This study demonstrates that rehabilitation professionals in Jordan have a generally positive attitude and foundational knowledge regarding TR. However, actual implementation remains constrained by technical, educational, and systemic barriers. Addressing these challenges through targeted training programs, improved infrastructure, and clear regulatory frameworks is advised to potentially enhance the effective integration of TR into routine clinical rehabilitation practice. Strengthening these areas may enhance access to rehabilitation services, improve continuity of care, and support the integration of TR into routine clinical practice, particularly in resource‐limited and geographically underserved settings.

## Author Contributions

**Mohammad Al-Wardat:** conceptualization, methodology, data curation, formal analysis, investigation, writing – original draft. **Khader Almhdawi:** conceptualization, methodology, supervision, validation, writing – review and editing. **Abidal Aziz Almomani** and **Mohammad Yabroudi:** data curation, formal analysis, visualization, writing – review and editing.

## Funding

No funding was received for this manuscript.

## Disclosure

The authors declare that they have no financial or personal relationship(s) that may have inappropriately influenced them in writing this article.

## Conflicts of Interest

The authors declare no conflicts of interest.

## Data Availability

The data that support the findings of this study are available on request from the corresponding author, M.A‐W.
